# Altered expression of IL-10 family cytokines in CRMO result in enhanced inflammasome activation

**DOI:** 10.1186/1546-0096-13-S1-O28

**Published:** 2015-09-28

**Authors:** S Hofmann, A Kubasch, A Rösen-Wolff, H Girschick, H Morbach, C Hedrich

**Affiliations:** 1 Universitätklinikum Carl Gustav Carus, TU Dresden, Children's Hospital, Pediatric Rheumatology and Immunology, Dresden, Germany; 2 Vivantes Klinikum-Friedrichshain, Children's Hospital, Berlin, Germany; 3 University of Würzburg, Children's Hospital, Würzburg, Germany

## Introduction

Chronic recurrent multifocal osteomyelitis (CRMO) is the most severe presentation of the autoinflammatory bone disorder chronic nonbacterial osteomyelitis (CNO). The pathophysiology of CNO remains to be determined. We recently demonstrated reduced activation of mitogen-activated protein kinases ERK1 and 2 in monocytes from CRMO patients responsible for impaired activation of the transcription factor signaling protein (Sp-)1. This resulted in failure to express the immuno-modulatory cytokine IL-10. The *IL10* gene, together with its homologues *IL19* and *IL20*, is organized in the 145 kb spanning *IL10* cytokine cluster on chromosome 1q32. In most cells, including monocytes, IL-10 cytokine family members are co-regulated in response to certain stimuli. IL-10 and IL-19 mainly have immune-modulating functions, while IL-20 acts as a pro-inflammatory cytokine contributing to inflammatory bone-loss. The NLRP3 inflammasome is a multi-protein complex forming in response to innate stimuli, subsequently mediating the cleavage and release of IL-1β. Enhanced inflammasome activation in IL-10 deficient mice was linked with bone-loss. Convincing evidence of this mechanism playing a role in CNO, however, is lacking.

## Objectives

The aim of our study was to determine i) IL-10 cytokine family expression patterns in CRMO monocytes, ii) molecular mechanisms underlying impaired cytokine expression, and iii) potential effects on inflammasome-dependent cytokine secretion.

## *Methods*

*Ex vivo* isolated monocytes from CRMO patients were cultured in the absence or presence of LPS. Expression patterns of cytokines were monitored on the transcriptional (mRNA) and protein level. Effects of impaired Sp-1 activation on cytokine expression were investigated through forced expression, chemical inhibition, or knock-down of Sp-1.

## *Results*

We saw reduced expression of anti-inflammatory cytokines IL-10 and IL-19 and unaffected expression of IL-20 in CRMO monocytes when compared to controls. We for the first time demonstrate Sp-1 recruitment to the *IL19* promoter, governing IL-19 expression in monocytes. Impaired expression of IL-10 and IL-19 in CRMO monocytes was caused by reduced binding of Sp-1 to regulatory regions. Expression of IL-20 was independent of Sp-1. Reduced IL-10 and IL-19 secretion from CRMO monocytes mediated increased activity of the NLRP3 inflammasome, as assessed by IL-1β secretion. Addition of recombinant IL-10 or IL-19 reversed these findings.

## *Conclusion*

Impaired activation of Sp-1 in monocytes from CRMO patients contributes to reduced expression of IL-10 and IL-19, resulting in an imbalance between pro- (IL-20) and anti-inflammatory IL-10 cytokine family members. Subsequently enhanced NLRP3 inflammasome activation results in IL-1β secretion which may in turn contribute to inflammatory bone-loss. A complete understanding of the molecular pathophysiology of CNO will aid in developing new disease biomarkers and therapeutic targets.

